# Chloroplastic *SaNADP-ME4* of C_3_–C_4_ Woody Desert Species *Salsola laricifolia* Confers Drought and Salt Stress Resistance to *Arabidopsis*

**DOI:** 10.3390/plants10091827

**Published:** 2021-09-03

**Authors:** Zhibin Wen, Yulan Wang, Chunlan Xia, Yuhui Zhang, Hongxiang Zhang

**Affiliations:** 1 State Key Laboratory of Desert and Oasis Ecology, Xinjiang Institute of Ecology and Geography, Chinese Academy of Sciences, Urumqi 830011, China; zhibinwen@ms.xjb.ac.cn (Z.W.); xywang_2018@126.com (Y.W.); xiachunlan18@mails.ucas.ac.cn (C.X.); zhangyuhui97@163.com (Y.Z.); 2 The Specimen Museum of Xinjiang Institute of Ecology and Geography, Chinese Academy of Sciences, Urumqi 830011, China; 3 University of Chinese Academy of Sciences, Beijing 100049, China

**Keywords:** *SaNADP-ME4*, C_3_–C_4_ intermediate species, abiotic stress, *Salsola laricifolia*

## Abstract

The NADP-malic enzyme (NADP-ME) catalyzes the reversible decarboxylation of L-malate to produce pyruvate, CO_2_, and NADPH in the presence of a bivalent cation. In addition, this enzyme plays crucial roles in plant developmental and environment responses, especially for the plastidic isoform. However, this isoform is less studied in C_3_–C_4_ intermediate species under drought and salt stresses than in C_3_ and C_4_ species. In the present study, we characterized *SaNADP-ME4* from the intermediate woody desert species *Salsola laricifolia*. *SaNADP-ME4* encoded a protein of 646 amino acids, which was found to be located in the chloroplasts based on confocal imaging. Quantitative real-time PCR analysis showed that *SaNADP-ME4* was highly expressed in leaves, followed by stems and roots, and *SaNADP-ME4* expression was improved and reached its maximum under the 200 mm mannitol and 100 mm NaCl treatments, respectively. *Arabidopsis* overexpressing *SaNADP-ME4* showed increased root length and fresh weight under mannitol and salt stress conditions at the seedling stage. In the adult stage, *SaNADP-ME4* could alleviate the decreased in chlorophyll contents and PSII photochemical efficiency, as well as improve the expression of superoxide dismutase, peroxidase, and pyrroline-5-carboxylate synthase genes to enhance reactive oxygen species scavenging capability and proline levels. Our results suggest that *SaNADP-ME4* overexpression in *Arabidopsis* increases drought and salt stress resistance.

## 1. Introduction

The NADP-malic enzyme (NADP-ME; EC 1.1.1.40) is an ancient and widely distributed enzyme involved in different metabolic pathways in bacteria, fungi, animals, and plants [1]. It catalyzes the reversible decarboxylation of L-malate to produce pyruvate, CO_2_, and NADPH in the presence of a bivalent cation [2]. Plant NADP-MEs are grouped into photosynthetic and non-photosynthetic isoforms. Photosynthetic NADP-MEs supply CO_2_ for carbon fixation in the bundle sheath cell chloroplasts of some C_4_ plants or the cytosol of some crassulacean acid metabolism (CAM) plants [2,3]. Additionally, non-photosynthetic NADP-MEs are distributed in the plastid or cytosol involved in various processes, including housekeeping roles such as the maintenances of cellular pH, generation of reductive power, regulation of guard cells, regulation of osmotic pressure for stomatal movement, fruit ripening, and response to multiple abiotic stresses [2,3,4,5,6,7,8,9].

The *NADP-ME* is encoded by a small multigene family, often including 2–5 *NADP-MEs*, and cytosolic and plastidic *NADP-ME* isoforms have also been described [4,10,11,12,13,14,15,16,17]. In C_3_ plants, there are four *NADP-MEs* in *Arabidopsis thaliana* and *Oryza sativa* consisting of three cytosolic and one plastidic *NADP-ME* [10,11], and there are five *NADP-MEs* in *Populus trichocarpa* consisting of three cytosolic and two putative plastidic *NADP-MEs* [16]. In C_4_ plants, there are five *NADP-MEs* in *Zea mays* including three cytosolic and two plastidic isoforms [4], and there are three *NADP-MEs* in *Flaveria trinervia* including one cytosolic and two plastidic isoforms [13,14]. Finally, there are three *NADP*-*MEs* in *Hydrilla verticillata* including two cytosolic and one plastidic *NADP-ME* [17]. In a CAM plant, *Aloe arborescens*, its *NADP-ME* family includes three genes, two cytosolic *NADP-MEs*, and one plastidic *NADP-ME* [12].

Suggestive data regarding the participation of *NADP-ME* in abiotic stress have been reported for several plant species [6,7], especially for the two common abiotic stress factors of drought and salinity [18,19]. In *Oryza sativa*, the expression level of plastidic *NADP-MEs* is elevated under all four stress conditions (mannitol, PEG, ABA, and NaCl) compared to that of controls. In contrast, the expression of three cytosolic *NADP-MEs* (*OscytME1*, *OscytME2*, and *OscytME3*) is induced by some stimuli and not by others. *OscytME1* is induced by PEG and NaCl; *OscytME2* is induced by mannitol, PEG, and NaCl; and *OscytME3* is induced by mannitol and NaCl [10]. Additionally, transgenic *Arabidopsis* plants overexpressing *OsNADP-ME2*, *OsNADP-ME3*, or *OsNADP-ME4* show resistance to salt and osmotic stresses [20,21,22]. In *Populus trichocarpa*, semi-quantitative RT-PCR analysis shows that five *NADP-MEs* respond to salt and osmotic stresses, and NaCl salts are found to significantly upregulate the transcripts of two putative plastidic NADP-MEs compared to another three cytosolic *NADP-MEs* [16]. In tobacco, PEG treatment or drought induces the increase of NADP-ME activity in leaves and increases transcript abundance of chloroplastic *NADP-ME* [15,23]. The constitutive expression of *ZmNADP-ME* in chloroplasts of tobacco plants confers drought tolerance by decreasing stomatal conductance and improving water use efficiency [24]. Restricted expression of chloroplastic *NADP-ME* (non-photosynthetic isoform) particularly in the guard and vascular companion cells of tobacco also enhances water use efficiency, and induces earlier flowering and shorter life cycle [15]. The overexpression of *SbNADP-ME* from sweet sorghum in *Arabidopsis* increases salt tolerance by improved proline content and alleviates PSII and PSI photoinhibition under salt stress by improving photosynthetic capacity [8]. These studies suggest that plastidic *NADP-ME* plays an important role in abiotic stresses [6].

However, little is known about the regulatory function of plastidic *NADP-MEs* from C_3_–C_4_ intermediate species under drought and salt stresses. C_3_–C_4_ intermediates have been identified in 56 species from 13 families of eudicots and monocots [25], which are different from C_3_ and C_4_ plants in structures and functions [26]. Additionally, C_3_–C_4_ lineages bridge the ecological gap to C_4_ photosynthesis [25]. Therefore, more *NADP-MEs* from C_3_–C_4_ intermediate species should be considered to better understand their functions and regulation mechanisms in plant tolerance to stress.

*Salsola laricifolia* is a woody desert species belonging to the family Amaranthaceae [27], and it is a typical C_3_–C_4_ intermediate species based on its anatomy, ultrastructure, and enzyme activity [28]. In previous studies, three *NADP-MEs* of *S. laricifolia* have been identified based on the transcriptome data of mixed leaf, stem, and root samples. Additionally, one of them (namely *SaNADP-ME4*) showed a higher similarity (74.7%) with *AtNADP-ME4* (a plastidic isoform from *Arabidopsis thaliana*, AT1G79750.1) than the other two *NADP-ME*s from *S. laricifolia*. In this study, we further cloned *SaNADP-ME4*. Based on confocal imaging, the *SaNADP-ME4* was found to be located in the chloroplasts. Additionally, the expression of *SaNADP-ME4* was significantly induced by mannitol and salt stresses in *S. laricifolia*, suggesting that it might be involved in these two stresses. Then, we transformed *SaNADP-ME4* into *Arabidopsis* and investigated the function of *SaNADP-ME4* during drought and salt stresses. Our study provides insight into the function of chloroplast *SaNADP-ME4* of C_3_–C_4_ intermediate species in abiotic stress resistance.

## 2. Results

### 2.1. The Protein SaNADP-ME4 Is Chloroplast-Localized

**Figure 1:**
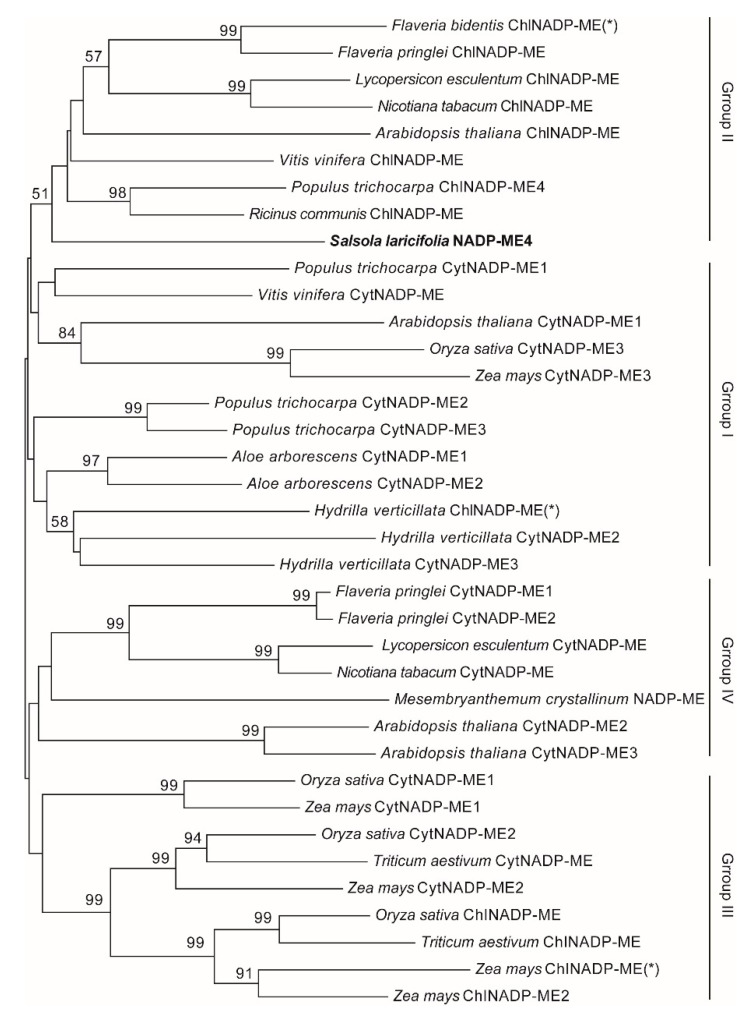
Phylogenetic analysis of *SaNADP-ME4* and other plant *NADP-MEs* via the neighbor joining method using MEGA 6.0 software. All plant *NADP-MEs* were classified into four groups: Group I (cytosolic eudicot types), Group II (plastidic eudicot types), Group III (monocot types), and Group IV (found in both eudicots and monocots). The C_4_ photosynthesis isoforms are marked with (*). Cyt, cytosolic; Chl, chloroplastic. The GenBank accession numbers and sources of the plant *NADP-ME*s are as follows: *Aloe arborescens* (CytNADP-ME1, BAA24950.1; CytNADP-ME2, BAA74735.1), *Arabidopsis thaliana* (CytNADP-ME1, AT2G19900.1; CytNADP-ME2, AT5G11670.1; CytNADP-ME3, AT5G25880.1; ChlNADP-ME, AT1G79750.1), *Flaveria bidentis* (ChlNADP-ME, AAW56450.1), *Flaveria pringlei* (CytNADP-ME1, AAK83073.1; CytNADP-ME2, AAK83074.1; ChlNADP-ME, P36444.1), *Hydrilla verticillata* (ChlNADP-ME, AY594687; CytNADP-ME2, AY594688; CytNADP-ME3, AY594689), *Lycopersicon esculentum* (ChlNADP-ME, AAB58727; CytNADP-ME, AAB58728), *Mesembryanthemum crystallinum* (NADP-ME, P37223.1), *Nicotiana tabacum* (ChlNADP-ME, DQ923119; CytNADP-ME, DQ923118), *Oryza sativa* (ChlNADP-ME, D16499; CytNADP-ME1, AB053295; CytNADP-ME2, AY435404; CytNADP-ME3, AY444338), *Populus trichocarpa* (CytNADP-ME1, 7461559; CytNADP-ME2, 7470734; CytNADP-ME3, 7476690; ChlNADP-ME, 7470579), *Ricinus communis* (ChlNADP-ME, AAF73006.1), *Triticum aestivum* (ChlNADP-ME, ABY25986.1; CytNADP-ME, ABW77317.1), *Vitis vinifera* (ChlNADP-ME, U67426; CytNADP-ME, L34836), and *Zea mays* (ChlNADP-ME1, J05130; ChlNADP-ME2, AY315822; CytNADP-ME1, AY104511; CytNADP-ME2, NM_001157493, CytNADP-ME 3, AY864063).

**Figure 2:**
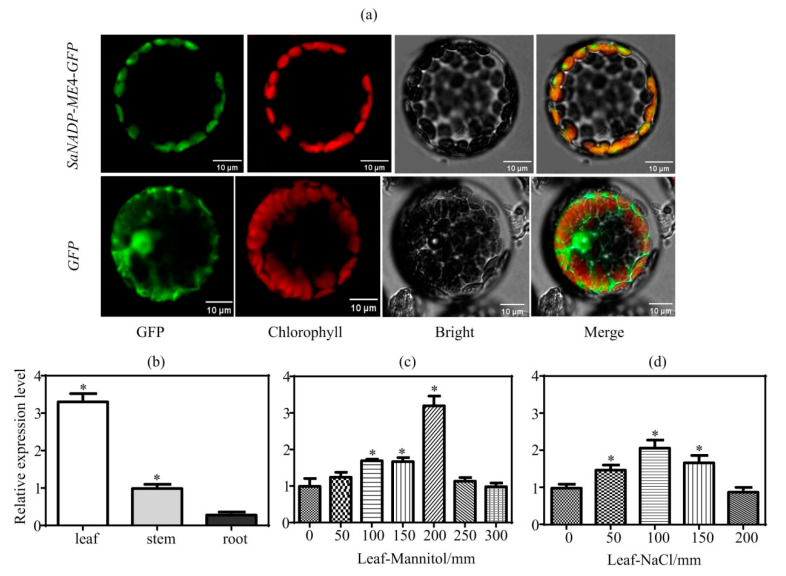
Subcellular localization of *SaNADP-ME4* and its expression in *Salsola laricifolia*. (**a**) *S*ubcellular localization of *SaNADP-ME4* in *Arabidopsis* protoplasts. *Arabidopsis* protoplasts isolated from 3-week-old leaves and transiently transformed with the *35S: SaNADP-ME4-GFP* construct or the *35S: GFP* as a control were analyzed by 16 h post transformation. Green and red fluorescence are indicative of the localization of the GFP protein and the chlorophyll, respectively. The images present GFP localization, chlorophyll localization, bright field, and a merge of the first three images. (**b**) The relative expression of *SaNADP-ME4* in leaves, stems, and roots of *S. laricifolia* based on RT-qPCR. The relative expression in other tissues was normalized by that in stems, which was set to 1.0. The samples were collected from 2-month-old *S. laricifolia* plants at 2 h after the beginning of a photoperiod. The *Sa18S* of *S. laricifolia* was used as a suitable reference gene for RT-qPCR in different tissues. (**c**,**d**) Expression analysis of *SaNADP-ME4* in the leaves of *S. laricifolia* under different levels of mannitol (**c**) and NaCl (**d**) stresses based on RT-qPCR. The relative expression in stress conditions was normalized by that in control condition, which was set to 1.0. Two-month-old plants were chosen. All treatments started at 2 h after the beginning of a photoperiod and lasted for 4 h. The *SaEF1-α* of *S. laricifolia* was used as a suitable reference gene for RT-qPCR in mannitol and salt stresses. Values are means ± SD (standard deviation). Asterisk indicates a significant difference at the *p* < 0.05 significance level, as determined with SPSS v. 19.0.

*SaNADP-ME4* was a 1941 bp ORF (GenBank No. MZ420664) that was predicted to encode a 646 amino acid protein. To gain insights into the position of *SaNADP-ME4*, the phylogenetic analyses of *SaNADP-ME4* and other plant *NADP-MEs* from 14 different plants (including *Aloe arborescens*, *Arabidopsis thaliana*, *Flaveria bidentis*, *Flaveria pringlei*, *Hydrilla verticillata*, *Lycopersicon esculentum*, *Mesembryanthemum crystallinum*, *Nicotiana tabacum*, *Oryza sativa*, *Populus trichocarpa*, *Ricinus communis*, *Triticum aestivum*, *Vitis vinifera*, and *Zea mays*) were carried out. All plant *NADP-MEs* were classified into four groups: Group I (cytosolic eudicot types), Group II (plastidic eudicot types), Group III (monocot types), and Group IV (found in both eudicots and monocots). *SaNADP-ME4* clustered with the plastidic eudicot *NADP-ME* group (Group II) (Figure 1). The *SaNADP-ME4-GFP* fusion gene and *GFP* under the control of the CaMV35S promoter were found to be transiently expressed in *Arabidopsis* protoplasts. Confocal imaging showed that *SaNADP-ME4-GFP* was distributed in the chloroplasts (Figure 2a).

A comparison of *SaNADP-ME4* and the other eight chloroplastic *NADP-ME* proteins in Group II showed that the predicted amino acid sequence of *SaNADP-ME4* had five conserved sites (Figure S1), a typical feature of *NADP-ME* proteins. Additionally, these five sites were conserved, as only one or two differences were found to exist in sites I and II (Figure S1). *SaNADP-ME4* was found to share 73.3% identity with *AtChlNADP-ME* (*Arabidopsis thaliana*, AT1G79750.1), 72.5% with *FbChlNADP-ME* (*Flaveria bidentis*, AAW56450.1), 73.5% with *FpChlNADP-ME* (*Flaveria pringlei*, P36444.1), 74.0% with *LeChlNADP-ME* (*Lycopersicon esculentum*, AAB58727), 73.7% with *NtChlNADP-ME* (*Nicotiana tabacum*, DQ923119), 75.6% with *PtChlNADP-ME* (*Populus trichocarpa*, 7470579), 76.3% with *RcChlNADP-ME* (*Ricinus communis*, AAF73006.1), and 76.5% with *VvChlNADP-ME* (*Vitis vinifera*, U67426), respectively.

### 2.2. Analysis of the Expression of SaNADP-ME4 in S. laricifolia

To determine the expression of *SaNADP-ME4* in *S. laricifolia*, the expression of *SaNADP-ME4* in three different tissues was determined using RT-qPCR. *SaNADP-ME4* was highly expressed in leaves, followed by the stems and roots (Figure 2b). Additionally, its protein localization was found to be in chloroplasts (Figure 2a). Therefore, the expression of *SaNADP-ME4* in leaves will be studied on abiotic stress.

For the investigation of whether *SaNADP-ME4* is involved in abiotic stress, the expression of *SaNADP-ME4* in response to mannitol and salt stresses was studied. Compared to the control, *SaNADP-ME4* expression was significantly enhanced to 1.7, 1.7, and 3.2 fold in 100, 150, and 200 mm mannitol treatments, respectively (Figure 2c), and it was enhanced to 1.5, 2.2, and 1.8 fold in 50, 100, and 150 mm NaCl treatments, respectively (Figure 2d). Taken together, these results indicate that the expression of *SaNADP-ME4* could be induced by mannitol and salt treatments.

### 2.3. Overexpression of SaNADP-ME4 Enhances Osmotic and Salt Tolerances at the Seedling Stage

Transgenic *Arabidopsis* plants with the overexpression of *SaNADP-ME4* were generated using an *Agrobacterium*-mediated transformation method for the analysis of the function of *SaNADP-ME4*. In total, 14 lines were generated. The transcript levels of *SaNADP-ME4* were first determined using RT-qPCR. The results showed that the exogenous *SaNADP-ME4* was significantly overexpressed in all transgenic lines, indicating that the overexpression lines had been successfully generated. Two transgenic lines (Lines 8 and 14) with relatively higher expression levels compared to other 12 lines were chosen for further studies on stress tolerance (Figure S2).

**Figure 3:**
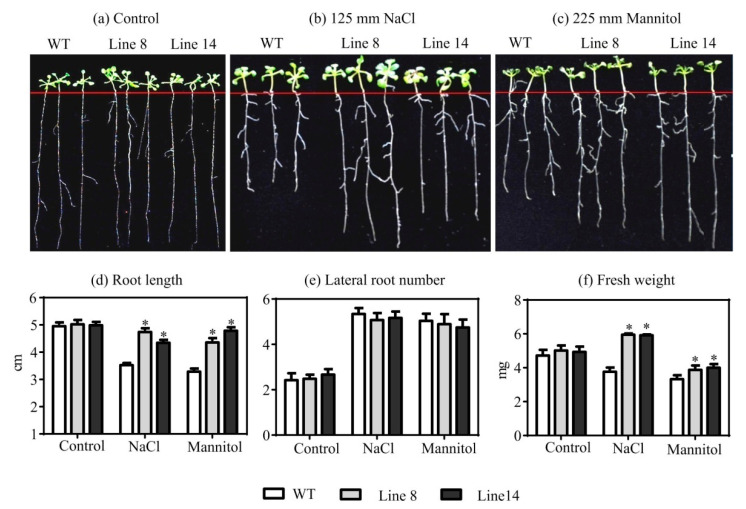
The seed germination percentage and growth of WT and two overexpressing lines of *Arabidopsis* plants under osmotic and salt stresses. (**a**–**c**) Phenotypes of WT and two overexpressing lines treated with an MS medium (control) (**a**), MS medium supplied with 125 mm NaCl (**b**), and an MS medium supplied with 225 mm mannitol (**c**) for 7 days. (**d**–**f**) Effect of different levels of mannitol and NaCl stresses on germination percentage (**d**), root length (**e**), and fresh weight (**f**) of WT and transgenic overexpressing *Arabidopsis* plants after 7 days. Values are means ± SD (standard deviation). Asterisk indicates a significant difference at the *p* < 0.05 significance level, as determined with SPSS v. 19.0.

No significant difference in the germination percentage, root length, and fresh weight of the WT and *Arabidopsis* overexpression lines was observed in the control conditions (Figure 3d–f). Additionally, the seed germination percentage showed that there were no obvious differences between the WT and transgenic lines under the 225 mm mannitol treatment. However, the seed germination percentage was inhibited by salt stress; that of the overexpressing Lines 8 and 14 were 63% and 71%, respectively, and it was 83% in the WT under the 125 mm NaCl treatment (*p* < 0.05) (Figure 3d). Under both the 125 mm NaCl and 225 mm mannitol treatments, transgenic seedlings had obvious longer root lengths and higher fresh weights than the WT seedlings, respectively (Figure 3e,f). However, there was no significant difference in lateral root number between the WT and transgenic seedlings under all treatments (Figure 3a–c). These results indicated that the overexpression of *SaNADP-ME4* enhanced the osmotic and salt stress resistance of transgenic *Arabidopsis* at the seedling stage.

### 2.4. Overexpression of SaNADP-ME4 Alleviates the Decreased in Chlorophyll Contents and PSII Photochemical Efficiency under Drought and Salt Stresses

**Figure 4:**
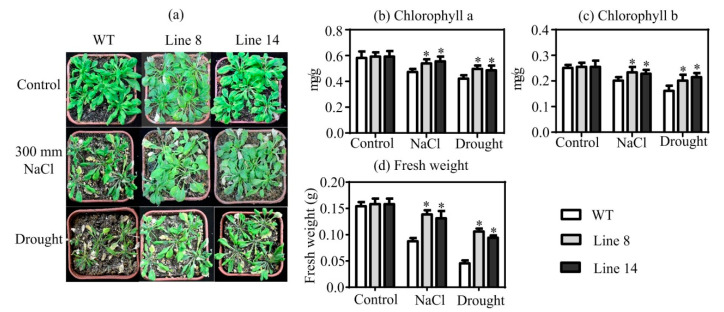
*SaNADP-ME4* confers NaCl and drought stress resistance. (**a**) The growth of WT and two overexpressing lines were compared under the control, 300 mm NaCl, and drought stress conditions for seven days. (**b**–**d**) The chlorophyll a content (**b**), chlorophyll b content (**c**), and fresh weight (**d**) were compared under the control, NaCl, and drought stress conditions. Values are means ± SD (standard deviation). Asterisk indicates a significant difference at the *p* < 0.05 significance level, as determined with SPSS v. 19.0.

Four-week-old WT and transgenic plants were compared under the control conditions, as well as drought and salt stresses. Under the control conditions, we observed no significant differences in chlorophyll a content, chlorophyll b content, and fresh weight (Figure 4b–d). Additionally, under drought and salt stresses, the overexpressing lines grew better than the WT line (Figure 4a). Though the chlorophyll a content, chlorophyll b content, and fresh weight were decreased compared to those in the control conditions, they were significantly higher than those in the WT plants under drought and salt stresses (Figure 4b–d).

We observed no significant differences in the PSII maximal photochemical efficiency (Fv/Fm) ratio and ETR (photosynthetic electron transportation rate) values of the WT and overexpressing *Arabidopsis* lines, respectively (Figure S3a,b). Though the Fv/Fm ratio and ETR values were decreased compared to those in the control conditions, they were significantly higher than those in the WT plants under drought and salt stresses (Figure S3a,b). The transgenic plants exhibited higher Y(II) (effective photochemical quantum yield of PSII) and Y(NPQ) (quantum yield of regulated energy dissipation of PSII)) values, as well as lower Y(NO) (quantum yield of non-regulated energy dissipation of PSII) values, relative to the WT line (*p* < 0.05) (Figure S3c–e). Under NaCl and drought stresses, Y(II) values were decreased compared to those in the control conditions, whereas Y(NPQ) and Y(NO) values were increased compared to those of the controls (Figure S3c–e). Under NaCl and drought stresses, the Y(NPQ) of the WT, Lane 8, and Lane 14 plants increased by 3.3 (3.3%), 11.8 (8.8%), and 6.1 (6.1%), respectively; the Y(NO) of WT, Lane 8, and Lane 14 increased by 8.0 (10.0%), 2.4 (9.7%), and 2.5 (7.5%), respectively. These results suggest that *SaNADP-ME4* confers significant drought and salt stress resistance to transgenic plants by alleviating their decreased PSII photochemical efficiency.

### 2.5. Overexpression of SaNADP-ME4 Decreases Oxidative Damages under Drought and Salt Stresses

**Figure 5:**
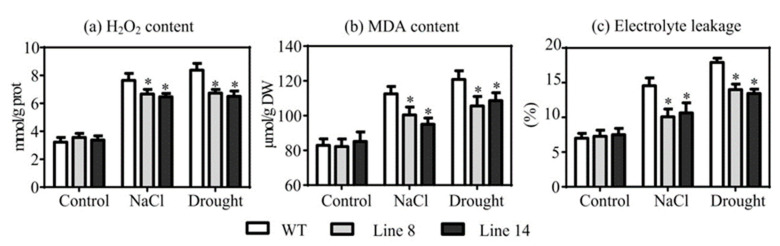
Comparison of hydrogen peroxide (H_2_O_2_) (**a**), malondialdehyde (MDA) content (**b**), and electrolyte leakage (**c**) of WT and two overexpressing lines under the control, 300 mm NaCl, and drought stress conditions. Values are means ± SD (standard deviation). Asterisk indicates a significant difference at the *p* < 0.05 significance level, as determined with SPSS v. 19.0.

To evaluate oxidative accumulation, hydrogen peroxide (H_2_O_2_) content was examined. The measurement of H_2_O_2_ content showed that two overexpressing lines accumulated less H_2_O_2_ than the WT plants under both drought and salt stresses (Figure 5a). Malondialdehyde (MDA) content and electrolyte leakage were used as indexes of membrane injury [29]. Under the control conditions, the MDA content and electrolyte leakage of all test lines did not greatly vary (Figure 5b,c). However, both the MDA content and electrolyte leakage were increased when exposed to salt and drought stress conditions. The two overexpressing lines accumulated less MDA than the WT line under both drought and salt stresses (Figure 5b). Consistently, electrolyte leakage was lower in the transgenic plants compared to the WT plants under both drought and salt stress conditions (Figure 5c). These results together suggest that *SaNADP-ME4* confers drought and salt stress resistance by decreasing oxidative damages.

### 2.6. Overexpression of SaNADP-ME4 Enhances Proline Biosynthesis under Drought and Salt Stresses

**Figure 6:**
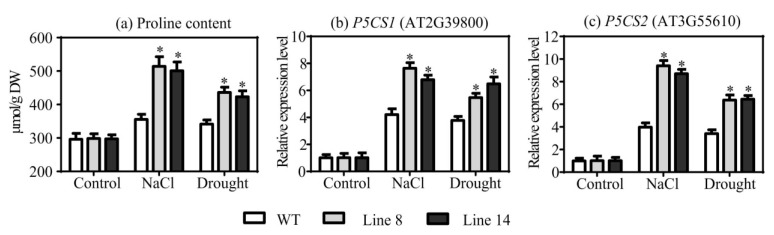
Analysis of proline biosynthesis affected by *SaNADP-ME4*. (**a**) Comparison of the proline content of WT and two overexpressing lines under the control, 300 mm NaCl, and drought stress conditions. (**b**,**c**) Analysis of the relative expression level of pyrroline-5-carboxylate synthase (*P5CS1*) (**b**) and *P5CS2* (**c**) under the control, NaCl, and drought stress conditions. The relative expression in stress conditions was normalized by that in control condition, which was set to 1.0. Values are means ± SD (standard deviation). Asterisks indicate a significant difference at the *p* < 0.05 significance level, as determined with SPSS v. 19.0.

To determine whether *SaNADP-ME4* is involved in adjusting osmotic potential, the proline contents were measured. Under the control conditions, the WT and two overexpression lines had similar proline contents (Figure 6a). However, when exposed to drought and salt stresses, all the studied lines displayed increased proline contents relative to those under the control conditions. In addition, compared to the WT plants, the overexpression lines accumulated significantly higher amounts of proline (Figure 6a). To further determine whether the overexpression of *SaNADP-ME4* could affect the relative expression level of proline biosynthesis-related genes, two genes—pyrroline-5-carboxylate synthase 1 (*P5CS1)* and *P5CS2*—were studied. There was no difference in the relative expression level of *P5CS1* and *P5CS2* between the WT and overexpression lines under the control conditions based on RT-qPCR. When exposed to drought and salt stresses, the relative expression levels of *P5CS1* and *P5CS2* were significantly higher in the two overexpression lines compared to those in the WT plants (Figure 6b,c).

### 2.7. Overexpression of SaNADP-ME4 Regulates of ROS Scavenging Capability under Drought and Salt Stresses

**Figure 7:**
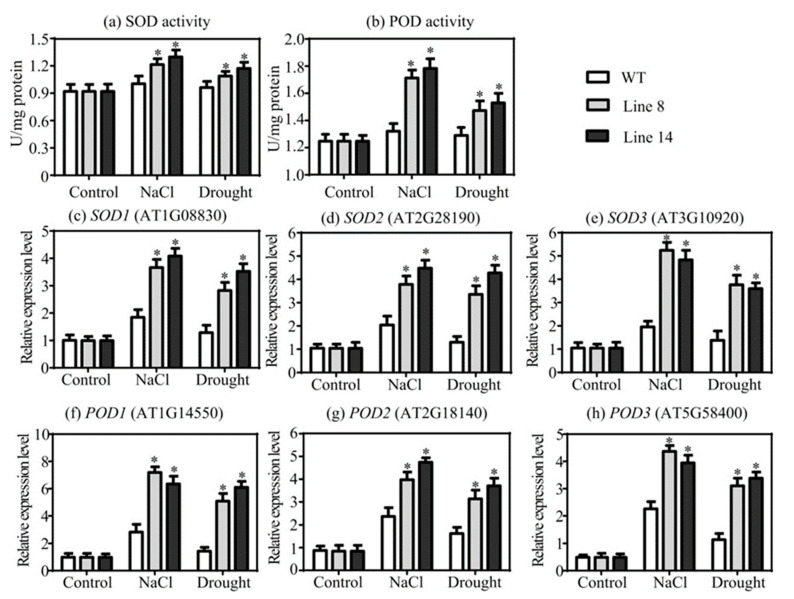
Analysis of reactive oxygen species (ROS) scavenging affected by *SaNADP-ME4*. (**a**,**b**) Measurement of superoxide dismutase (SOD) (**a**) and peroxidase (POD) (**b**) activities among WT and two overexpression lines under the control, 300 mm NaCl, and drought stress conditions. (**c**–**h**) Analysis of the relative expression level of *SOD1* (**c**), *SOD2* (**d**), *SOD3* (**e**), *POD1* (**f**), *POD2* (**g**), and *POD3* (**h**) under the control, NaCl, and drought stress conditions. The relative expression in stress conditions was normalized by that in control condition, which was set to 1.0. Values are means ± SD (standard deviation). Asterisks indicate a significant difference at the *p* < 0.05 significance level, as determined with SPSS v. 19.0.

The activities of two main reactive oxygen species (ROS) scavenging enzymes including superoxide dismutase (SOD) and peroxidase (POD) were analyzed in transgenic and WT plants under drought and salt stress conditions. Similar changes were observed in the two overexpressing lines, since similar levels of SOD and POD activities were detected in the transgenic and WT plants under the control conditions, but we also observed substantial increases in SOD and POD activities in the drought-treated and salt-treated transgenic lines compared to the WT plants (Figure 7a,b). To further determine whether the varied SOD and POD activities were caused by changes in the expression of *SOD* and *POD* genes, the relative expression levels of six related genes were examined. There were no differences in the relative expression levels of *SOD1*, *SOD2*, *SOD3*, *POD1*, *POD2*, and *POD3* among all studied lines under the control conditions. When exposed to drought and salt stresses, the relative expression levels of these six genes were significantly higher in the two overexpressing lines compared to those in the WT plants (Figure 7c–h).

### 2.8. Overexpression of SaNADP-ME4 Increases of the Total NADP-ME Activity under Drought and Salt Stresses

The total NADP-ME activity in the leaf protein extracts of the overexpressing plants was examined. The two overexpressing lines showed ~2 fold increases of NADP-ME activity relative to the WT plants under the control conditions. Additionally, when exposed to drought and salt stresses, all the studied lines displayed increased NADP-ME activity relative to those under the control conditions. In addition, compared to the WT plants, the overexpression lines accumulated significantly higher NADP-ME activities under drought and salt stresses (Figure S4).

## 3. Discussion

*Salsola laricifolia* is a typical desert C_3_–C_4_ intermediate species [28]. In the present work, we isolated the chloroplastic *SaNADP-ME4* and transformed it into *Arabidopsis* to investigate its function under drought and salt stresses. *SaNADP-ME4* was found to encode a protein of 646 amino acids, to be located in the chloroplasts based on confocal imaging (Figure 2a), and to have amino acid sequences with five conserved sites (sites I–V) (Figure S1) common to plant *NADP-ME*s [3]. Additionally, the expression of *SaNADP-ME4* was not found to be tissue-specific (Figure 2b), as shown in *Arabidopsis thaliana NADP-ME4* (chloroplastic type) [11], *Populus trichocarpa NADP-ME4,* and *NADP-ME5* (putative plastidic types) [16]. Plant *NADP-ME*s have been divided into four groups: Group I (cytosolic dicot types), Group II (plastidic dicot types), Group III (monocot types), and Group IV (both eudicots and monocots) [3,11,16,30]. The present results showed that *SaNADP-ME4* clustered with the plastidic eudicot *NADP-ME* group (Group II) (Figure 1). Additionally, the alignment of the protein sequences of *SaNADP-ME4* and the other eight chloroplastic *NADP-MEs* in Group II showed that *SaNADP-ME4* had higher chloroplastic *NADP-ME* sequences from seven C_3_ plants than the photosynthetic isoform from the C_4_ plant *Flaveria bidentis* (*FbChlNADP-ME*) (see Section 2.1).

In the present study, the analysis of *SaNADP-ME4* transcripts in the leaves of *S. laricifolia* under different mannitol and salt treatments showed that the highest transcript level was attained under the 200 mm mannitol and 100 mm NaCl treatments (Figure 2c,d), suggesting that *SaNADP-ME4* may be involved in osmotic and salt responses. Furthermore, *SaNADP-ME4* can positively regulate the osmotic and salt stress tolerance of *Arabidopsis* overexpression plants in both the seedling and adult stages. At the seedling stage, the root length of the WT and overexpression lines were inhibited in both the 125 mm NaCl and 225 mm mannitol treatments compared to that of all studied lines in the control conditions. Additionally, the degree of inhibition in the WT line was significantly higher than that of the overexpression lines (Figure 3e). Similar changes of fresh weight were found in all studied lines under the mannitol treatment. However, under NaCl stress, the fresh weight of the overexpression lines was higher than that in the control conditions (Figure 3f). These results suggest that the overexpression of *SaNADP-ME4* increases plant osmotic and salt stress resistance to some extent at the seedling stage.

Previous studies have described the plastidic *NADP-ME* as a stress-responsive enzyme [4,8,15]. In this present study, the overexpression of *SaNADP-ME4* conferred drought and salt stress resistance to *Arabidopsis* in the adult stage. *SaNADP-ME4*-overexpressed plants showed better growth phenotypes and possessed greater fresh weights than the WT line under the drought and salt stress conditions (Figure 4a). The chlorophyll content and florescence parameters of the WT and overexpression lines were compared under NaCl and drought treatments in all studied lines (Figure 4b,c and Figure S3). Chlorophyll content is an important indicator of photosynthetic capacity and can reflect it to some degree [31]. Under drought and salt stress conditions, the chlorophyll a and b contents of the *Arabidopsis* plants were reduced. In addition, the chlorophyll a and b contents decreased less in the overexpression lines than in the WT line (Figure 4b,c). There is usually a close connection between the chlorophyll content and photochemical efficiency of PSII [31]. A higher chlorophyll a content was found to result in higher photochemical efficiency values of PSII in *SaNADP-ME4* overexpression lines (Figure S3a–d).

The model of Kramer et al. [32] suggests that the quantum yield of PS II consists of Y(II), Y(NPQ), and Y(NO), which represent light energy absorbed by PSII used in photochemical reactions, heat dissipation via safe light-regulated quenching, and heat dissipation via harmful non-regulated non-photochemical quenching in PSII, respectively. In the present study, higher Y(II) and Y(NPQ) and lower Y(NO) values relative to the WT line were exhibited in the control condition, suggesting that the overexpression levels led to a higher photochemical efficiency of PSII. Under salt and drought stresses, Y(II) values decreased to a lesser degree than that of the WT line (Figure S3c), suggesting that the decrease in light energy used in photochemical reaction was alleviated in the overexpression lines compared to the WT line. The heat dissipation through the NPQ of chlorophyll fluorescence plays an important role in protecting plants from stresses. Additionally, Y(NO) is an important index of light damage [32,33]. The present results showed that the extent of increase in the Y(NPQ) and Y(NO) of overexpression lines was different under NaCl and drought stresses, i.e., to a greater extent in Y(NPQ) and a lesser extent in Y(NO) compared to the WT line (Figure S3d,e), suggesting that the extent of damage from excess light energy was less in the overexpression lines; as a result, the Fv/Fm and ETR were higher (Figure S3a,b). These results suggest that the decrease of PS II photochemical efficiency was alleviated by the overexpression of *SaNADP-ME4* in *Arabidopsis*. A similar study showed that the overexpression of *SbNADP-ME* from sweet sorghum in *Arabidopsis* alleviates the decreased in PSII and PSI photoinhibition under salt stress in the seedling stage [8].

When exposed to various abiotic stresses, the excess production of ROS can cause oxidative damage to cellular components, such as membranes, DNA, protein, and lipids [34]. Therefore, it is important for plants to control ROS at a suitable level. The activities of two main ROS scavenging enzymes (SOD and POD) were analyzed in all studied lines under drought and salt stress conditions. The present results showed that under drought and salt stress conditions, the activities of SOD and POD were significantly improved in transgenic plants relative to those in the WT plants (Figure 7a,b), as did in the expression of the *SOD* and *POD* genes (Figure 7c–h). Additionally, the H_2_O_2_ content in the transgenic plants was greatly reduced compared to the WT plants (Figure 5a). In general, these results showed that *SaNADP-ME4* positively affected the regulation of the *SOD* and *POD* genes, thus contributing to the increased SOD and POD activities and resulting in decreased H_2_O_2_ levels and enhanced drought and salt stress tolerance.

The present results showed that the total NADP-ME activity in the WT and transgenic plants was significantly increased under drought and salt stress conditions (Figure S4). Both the PEG treatment and drought stress caused increases in NADP-ME activity in tomato leaves and chloroplast *NADP-ME* expression [15,23]. The NADP-ME, especially the chloroplast NADP-ME, plays an important role in drought stress sensing and confers excessive ROS detoxification in chloroplasts [6]. The NADP-ME, which provides NADPH, is essential for antioxidant functions [35]. The NADP-ME and other NADPH-producing dehydrogenases such as glucose-6-phosphate dehydrogenase (G6PDH), isocitrate dehydrogenase (ICDH), and ferredoxin-NADP reductase (FNR), which recycle NADPH, are necessary for protection against oxidative damages in olive plants [35]. NADPH is also an important molecule in the redox balance of a cell [6,35,36]. Redox regulation and ROS metabolism are interlinked and interconnect mitochondria, chloroplast, and other organelles to a wider cellular redox-network. The redox state of NAD(P)H is one of the factors that influences that network [36]. Additionally, new tools are available for monitoring NADPH dynamics and manipulating NADP^+^/NADPH in live cells [37,38]. Therefore, NADPH may be important in revealing the function of *SaNADP-ME4* in ROS scavenging under stress.

Proline is an important oxmolyte when exposed to various abiotic stresses, acting to maintain redox balance and radically scavenging to protect plant cells [39]. In the present study, both proline content (Figure 6a) and the expression of proline biosynthesis genes (*P5CS1* and *P5CS2*) in the WT and transgenic plants were greatly increased under drought and salt stress conditions (Figure 6b,c). Therefore, the increase in proline content caused by *SaNADP-ME4* may enable positive adjustments in *Arabidopsis* under drought and salt stress conditions. The overexpression of *SbNADP-ME* from sweet sorghum in *Arabidopsis* was found to increase proline content and result in salt resistance [8]. The induced NADP-ME activity linked to drought and salt stresses (Figure S4) was probably involved in providing particular NAD(P)H, not only to supply reducing power for the synthesis of proline but also in general for the basal metabolism of plants—in particular, under stress [36,40].

## 4. Materials and Methods

### 4.1. Plant Materials and Growth Conditions

Seeds of *Salsola laricifolia* were collected from Toli City (Xinjiang, China). Seeds were stored at 4 °C before being germinated on moist paper at room temperature, and they were then placed in a growth chamber (a relative humidity of approximately 40–50%, day/night temperatures of 25/18 °C, a 14/10 h light/night photoperiod, and an irradiance of 400 μmol photons m^–2^s^–1^). Then, two-week-old seedlings were transplanted to 9 cm diameter pots with a Hoagland solution. Each pot was supplemented with a float with eight holes and contained eight plants. Irradiance was increased to 1000 μmol photons m^–2^s^–1^ [41] until two-month-old plants were chosen. The Hoagland solution was changed every week.

The *Arabidopsis thaliana* L. Col-0 ecotype was used as the genetic background/wild type (WT) for the transgenic plants generated in this study. Plants were grown in a chamber with standard growth conditions (a relative humidity of approximately 60–70%, day/night temperatures of 22/20 °C, a 16/8 h light/night photoperiod, and an irradiance of 400 μmol photons m^−2^s^−1^).

### 4.2. Sequence and Phylogenetic Analysis

Multiple alignments of complete predicted amino acid sequences of *NADP-MEs* from 14 different plants accessible from NCBI public databases were performed using ClusterW 2. The phylogenetic tree was constructed with the neighbor joining method using the MEGA 6.0 program [42]. Usually, there were five sites within the amino acid sequences of the *NADP-ME*s on the basis of the C_4_-*NADP-ME* from maize: site I (VYTPTVGEACQKYG), site II (IQVIVVTDGERILGLGDLGCQGMGIPVGKL), site III (QFEDFANHNAF), site IV (FNDDIQGTASVVL), and site V (LFLGAGEAGTGIAEL) [2]. To ascertain whether *SaNADP-ME4* had these five conserved sites, the protein sequences of *SaNADP-ME4* and eight other chloroplastic *NADP-MEs* were aligned: *AtChlNADP-ME*, *FbChlNADP-ME*; *FpChlNADP-ME*, *LeChlNADP-ME*, *NtChlNADP-ME*, *PtChlNADP-ME*, *RcChlNADP-ME*, and *VvChlNADP-ME*.

### 4.3. Subcellular Localization Analysis

The PCR products corresponding to the full coding sequence (without stop codon) were first cloned in pMD18-T using gene-specific primers containing an *SmaI* restriction site and then fused with the N-terminus of the green fluorescent protein (GFP) reporter, the expression of which was driven by the CaMV 35S promoter in the pBI121 vector. The primers for vector construction were designed with Primer Premier 5 were: pBI121-GFP-F: TTTCATTTGGAGAGAACACG; pBI121-GFP-R: CGACCAGGATGGGCACCAC; pBI121(in-fusion)-*SaNADP-ME4*-F: ACTCTAGACTGGTACCCATGATCTCTCTTCAAA; and pBI121(in-fusion)-*SaNADP-ME4*-R: CTAGTCAGTCGACCCTCACCGGTAGCTTCTGT. The vector sequences were underlined.

The *SaNADP-ME4-GFP* construct was used to transform the *Arabidopsis* protoplasts obtained from the fresh leaf tissue of 3-week-old plants for transient transformation. Protoplast isolation from *A. thaliana* leaves, as well as a transient expression assay, was conducted as previously described [43,44]. GFP and chlorophyll were excited with 488 and 633 nm laser lines, respectively, using a laser confocal microscope (Zeiss LSM 800, Jena, Germany) equipped with Zen software to process the image. The fluorescence signals were detected at 500–530 nm for GFP and at 650–750 nm for chlorophyll after exciting at 488 and 633 nm, respectively. Representative protoplasts from at least two independent experiments are shown on the same scale, including their merged and light fields.

### 4.4. Expression Analysis of SaNADP-ME4 in S. laricifolia under Different Tissues, Mannitol and NaCl Stresses

Two-month-old plants were chosen. All treatments started at 2 h after the beginning of a photoperiod. For different tissues, leaves, stems, and roots were all collected, immediately frozen in liquid nitrogen, and stored at −80 °C until use. For mannitol and NaCl stresses, a Hoagland solution with 0, 50, 100, 150, 200, 250, and 300 mm mannitol or 0, 50, 100, 150, and 200 mm NaCl was applied for 4 h, and then leaves were collected, immediately frozen in liquid nitrogen, and stored at −80 °C until use. Each treatment had four biological replicates.

Total RNA was isolated from 100 mg of frozen samples using a TransZol reagent (Transgen Biotech, Beijing, China) (four independent extractions for each treatment) following the manufacturer’s instructions. The RNA samples with absorption ratios of A_260_/A_280_ = 1.8–2.2 and A_260_/A_230_ higher than 2.0 were used for subsequent complementary DNA synthesis. The first-strand cDNA was synthesized using a Reversal Transcription Reagent Kit (Takara, Tokyo, Japan), following the manufacturer’s instructions. RT-qPCR was performed with a CFX96 Real-Time PCR detection system (Bio-Rad, Hercules, CA, USA) using SYBR Premix Ex Taq^™^ (Takara, Tokyo, Japan). The *Sa18S* and *SaEF1-α* of *S. laricifolia* were used as suitable reference genes for RT-qPCR in different tissues and different stresses, respectively [41]. The conditions for the amplification of RT-qPCR were: polymerase activation at 95 °C for 30 s and 40 cycles, with each cycle comprising 95 °C for 30 s, 55 °C for 10 s, and 72 °C for 15 s. All samples were run with two technical replicates, and three no template controls (replacing the template product with PCR-grade water) were included in every run to monitor possible DNA contamination. The relative expression of the detected genes was calculated using the 2^−ΔΔCt^ method [45]. The primer pairs were designed with Primer Premier 5 as follows: *SaNADP-ME4*-F*:* GTTGTTACTGATGGTGAGCGGATTT; *SaNADP-ME4*-R*:* GGACGAATGCCACCAAGAGC; *Sa18S*-F: GGGCATTCGTATTTCATAGTCA; *Sa18S*-R: CGGCATCGTTTATGGTTGA; *SaEF1-α*-F: TCAGTTTGGTGGTTATTGGACA; and *SaEF1-α*-R: ACCTCTTGTTCATCTCAGCAGC.

### 4.5. Generation of SaNADP-ME4 Overexpressing Arabidopsis

The *SaNADP-ME4-GFP* construct was introduced into the WT *Arabidopsis* plant via the floral dip method [46]. T1–T3 generation seeds of transgenic plants were selected on a Murashige and Skoog (MS) medium containing 50 μg/mL of kanamycin. The kanamycin-resistant T1 seedlings were tested by PCR analysis and sequencing, and transgenic T3 homozygous lines were chosen for further studies based on their high expression levels of *SaNADP-ME4*. *α-tubulin* (AT1G50010) was used as a suitable reference for RT-qPCR in *SaNADP-ME4* expression and different stresses [47]. The primers used in this experiment are listed in Table S1.

### 4.6. Assessment of the Mannitol and NaCl Stress Tolerance of Transgenic Arabidopsis at Germination Stage

For the germination stage, both the WT and transgenic T3 seeds were sterilized and then placed on MS medium plates (control) or MS medium plates supplied with either 125 mm NaCl or 225 mm mannitol. These plates were placed in refrigerator at 4 °C for 48 h and then on chamber benches with the standard growth conditions of *A. thaliana*. For each plate, at least 40 seeds were placed. Germination percentages were calculated after 7 days. Each treatment had four biological replicates.

### 4.7. Assessment of Mannitol and NaCl Stress Tolerance of Transgenic Arabidopsis at Seedling Stage

For the seedling stage, 7-day-old seedlings of WT and transgenic *Arabidopsis* cultured on the MS medium were transferred on the MS medium (control) or the MS medium supplied with either 125 mm NaCl or 225 mm mannitol, and then they were maintained for 7 days after a visible phenotype became evident. More than 30 seedlings of WT and each transgenic line were used for calculating the root length, lateral root number, and fresh weight.

### 4.8. Assessment of Drought and NaCl Stress Tolerance of Transgenic Arabidopsis at Adult Stage

Four-week-old plants from the WT and transgenic *Arabidopsis* cultured on soil (nutritive soil:vermiculite:perlite = 3:1:1, *v*/*v*) were chosen. For NaCl stress, *Arabidopsis* plants were watered with 300 mm NaCl and maintained for 7 days after a visible phenotype became evident. For drought stress, *Arabidopsis* plants were not provided with water and were maintained for 7 days after a visible phenotype became evident. For the fresh weight, 10 plants of the WT and each transgenic line under each condition were used.

For chlorophyll content, samples of fresh leaves were homogenized in a mortar and suspended in 3 mL of 96% (*v*/*v*) ethanol per 50 mg of tissue. After incubation in the dark until no green was visible, the extracts were centrifuged for 5 min at 10,000× *g*; then, the supernatants were used to measured absorbance at 665 and 649 nm to obtain the concentrations of chlorophyll a and b [48]. Chlorophyll fluorescence was measured using PAM-2500 (Walz, Würzburg, Germany). Initially, plant material was incubated in the dark for 30 min. Minimal fluorescence (Fo) and maximum (Fm) florescence were measured with very weak red light (0.1 μmoL photons m^−2^s^−1^) and saturating light pulse (8000 μmol photons m^−2^s^−1^), respectively. The Fv/Fm ratio was determined as (Fm−Fo)/Fm. For ETR, Y(II), Y(NPQ), and Y(NO) values were obtained at the 500 μmol photons m^−2^s^−1^ light intensity. 

Electrolyte leakage was assessed by ion leakage analysis, as previously described [49]. For enzyme extracts and assays for H_2_O_2_ content, MDA content, the total SOD activity, and POD activity, fresh leaves (0.3 g of fresh weight (FW)) were ground with an ice-cold 0.1 mol L^−1^ potassium phosphate buffer (pH 7.4), as suggested by the kit manufacturer (Nanjing Jiancheng Bioengineering Institute, China) (http://www.njjcbio.com/contents.asp?cid=2&wid=2&id=756) (accessed on 16 May 2019) and published literature [47,50,51]. Then, the extracts were centrifuged for 10 min at 8000× *g* (4 °C) and the supernatants were measured using assay kits according to the manufacturer’s instructions (kit no. A064, A003-1, A001-1, A084-3; Nanjing Jiancheng Bioengineering Institute, China). The extractions and assays were all based on the manufacturers’ instruction. H_2_O_2_ content was determined using the method described by Jana and Choudhuri [52]. Briefly, 1 mL of supernatant was thoroughly mixed with 1 mL of 0.1% titanium sulphate in 20% H_2_SO_4_ (*v*/*v*), and the mixture was then centrifuged at 6000× *g* for 15 min at room temperature. The absorbance of the yellow color of the supernatant was measured at 410 nm. The measurement of MDA content was based on the thiobarbituric acid (TBA) method [53]. Briefly, after mixing trichloroacetic acid with the homogenate and centrifuging, a supernatant was obtained and TBA was added. The developed red color of the resulting reaction was measured at 532 nm with a spectrophotometer. The total activity of SOD was determined by measuring the inhibiting rate of the enzyme to the O_2_^−^·produced by the xanthine morpholine with xanthine oxidase using the SOD assay kit [54]. The POD activity was measured based on the change of absorbance at 420 nm by catalyzing H_2_O_2_ [55]. The quantitation of total protein was measured with the bicinchoninic acid (BCA) method [56] using an assay kit (no. A045; Nanjing Jiancheng Bioengineering Institute, China). Each treatment had four biological replicates. Proline content was measured using an acid-ninhydrin reagent and acetic acid [57]. Briefly, fresh leaves were ground with an ice-cold extraction solution provided in an assay kit (no. A107-1; Nanjing Jiancheng Bioengineering Institute, Nanjing, China). Well-mixed solutions were boiled at 100 °C for 30 min. After cooling to room temperature, the proline levels of samples were calculated at 520 nm absorbance.

Additionally, the expression of eight related genes—*P5CS1* (AT2G39800), *P5CS2* (AT3G55610), *SOD1* (AT1G08830), *SOD2* (AT2G28190), *SOD3* (AT3G10920), *POD1* (AT1G14550), *POD2* (AT2G18140), and *POD3* (AT5G58400)—was evaluated by RT-qPCR. *α-tubulin* was used as a suitable reference for RT-qPCR. The conditions for the amplification of RT-qPCR were: polymerase activation at 95 °C for 30 s and 40 cycles, which each cycle comprising 95 °C for 30 s, 55–60 °C for 10 s, and 72 °C for 15 s. The primers for this experiment are listed in Table S1.

The activities of the total NADP-ME activity were determined in supernatant according to Gerrard Wheeler et al. [11], and frozen leaves were used. NADP-ME activity was assayed using a mixture of 50 mm Tris-HCl (pH 7.5), 0.5 mm NADP, 10 mm malate, 10 mm MgCl_2_, and 10 μL of an extract containing the enzyme [11]. Each treatment had four biological replicates.

### 4.9. Statistical Analysis

All statistical tests were performed with SPSS v. 19.0 (SPSS Inc., Chicago, IL, USA) using an ANOVA, and differences were considered statistically significant at * *p* < 0.05.

## 5. Conclusions

In summary, we demonstrated that the expression of *SaNADP-ME4* in the C_3_–C_4_ woody desert plant *S. laricifolia* is induced by drought and salt stresses. The overexpression of *SaNADP-ME4* increased root length and fresh weight under mannitol and salt stress conditions at the seedling stage. At the adult stage, the overexpression of *SaNADP-ME4* in *Arabidopsis* conferred drought and salt stress tolerance by alleviating decreases in chlorophyll content and PSII photochemical efficiency, as well as be activating ROS scavenging capability and osmotic adjustments. These findings provide useful information for the function of the chloroplast *SaNADP-ME4* of intermediate species in abiotic stress resistance.

## Data Availability

The data presented in this study is available in the article.

## Supplementary Materials

The following are available online at https://www.mdpi.com/article/10.3390/plants10091827/s1. Figure S1: Alignment of the predicted amino acid sequences between *SaNADP-ME4* and eight other chloroplastic *NADP-MEs*. Conserved sequences site (I–V) were found in all *NADP-MEs*. The access numbers to GenBank of each sequence are: *AtChlNADP-ME* (AT1G79750.1), *FbChlNADP-ME* (AAW56450.1), *FpChlNADP-ME* (P36444.1), *LeChlNADP-ME* (AAB58727), *NtChlNADP-ME* (DQ923119), *PtChlNADP-ME* (7470579), *RcChlNADP-ME* (AAF73006.1), and *VvChlNADP-ME* (U67426); Figure S2: Molecular identification of the *SaNADP-ME4* transgenic *Arabidopsis* lines. (**a**) Genomic DNA PCR of overexpression lines, (**b**) the relative expression level of *SaNADP-ME4* in the overexpression lines; Figure S3: Comparison of the Fv/Fm (PSII maximal photochemical efficiency) (**a**), ETR (photosynthetic electron transportation rate) (**b**), Y(II) (effective photochemical quantum yield of PSII) (**c**), Y(NPQ) (quantum yield of regulated energy dissipation of PSII) (**d**), and Y(NO) (quantum yield of non-regulated energy dissipation of PSII) (**e**) of WT and two overexpressing lines under the control, NaCl, and drought stress conditions. Values are means ± SD (standard deviation). Asterisk indicates a significant difference at the *p* < 0.05 significance level, as determined with SPSS v. 19.0; Figure S4: Analysis of the total NADP-ME activity affected by *SaNADP-ME4*. Values are means ± SD (standard deviation). Asterisks indicate a significant difference at the *p* < 0.05 significance level, as determined with SPSS v. 19.0.
