# Supplementary material for: Chloroplastic SaNADP-ME4 of C3–C4 Woody Desert Species Salsola laricifolia Confers Drought and Salt Stress Resistance to Arabidopsis
Source: Plants (Basel). 2021 Sep 3;10(9):1827. doi: 10.3390/plants10091827 (PMC8471237; doi:10.3390/plants10091827)
Supplement: Supplementary file 1 [file plants-10-01827-s001.zip › Table S1.pdf]

Table S1 List of primers for RT-qPCR

Gene name	GenBank accession number	Primers (5' to 3')
P5CS1	AT2G39800	F: ACCAGAAGCACGGTCATTC<br>R: CCATCTGAGAATCTTGTG
P5CS2	AT3G55610	F: ATGATCTTATTTATGTTCTGC<br>R: CACTACTTCCGTCACTAT
SOD1	AT1G08830	F: ATGTCTACTGGTCCACATTTC<br>R: ATGGCCTCCCTTTCCGAG
SOD2	AT2G28190	F: ATGTATCTCAACAGGACCAC<br>R: AGTGGTCAGACTAAGCTC
SOD3	AT3G10920	F: AGTGAAGGTGGTGGAGAGC<br>G: CATCTATACCCACCAGAG
POD1	AT1G14550	F: CCATAGGACAATCTCAATGC<br>R: TGATCGGTTACTAATAG
POD2	AT2G18140	F: TCCGGGAGCCACACCATTGG<br>R: TGGTCGGAATTCAACAG
POD3	AT5G58400	F: GGCAAGCCAGGTGCGTCAC<br>R: CCGGCTGTAGGATACGAC
α-tubulin	AT1G50010	F: GATGTACCGTGGTGATGTC<br>R: GAGCCTCTGAAAATTCTCC
