# Supplementary figures and images for: Chloroplastic SaNADP-ME4 of C3–C4 Woody Desert Species Salsola laricifolia Confers Drought and Salt Stress Resistance to Arabidopsis
Source: Plants (Basel). 2021 Sep 3;10(9):1827. doi: 10.3390/plants10091827 (PMC8471237; doi:10.3390/plants10091827)

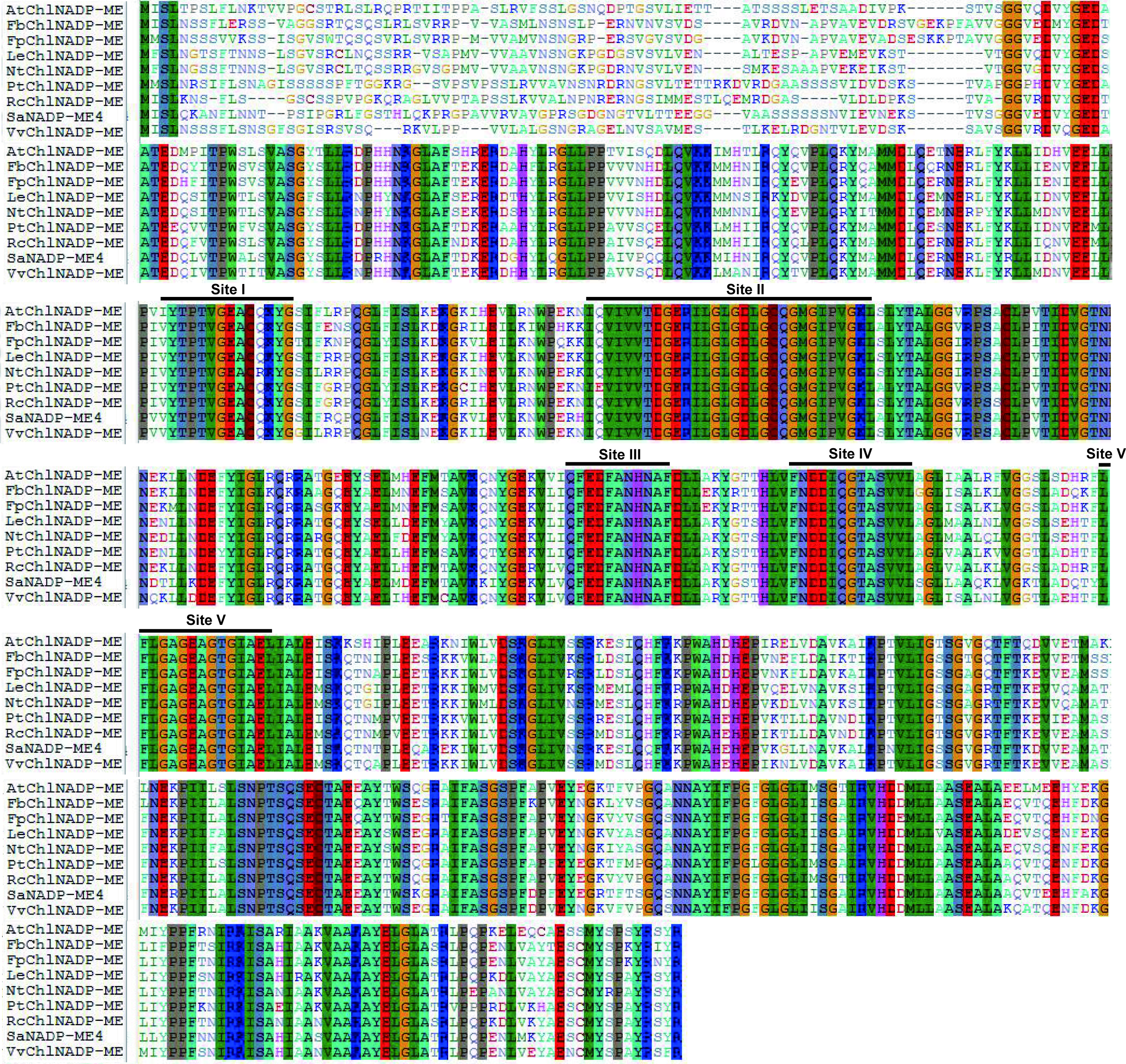

Supplement: Supplementary file 1 [file plants-10-01827-s001.zip › Figure S1.jpg]

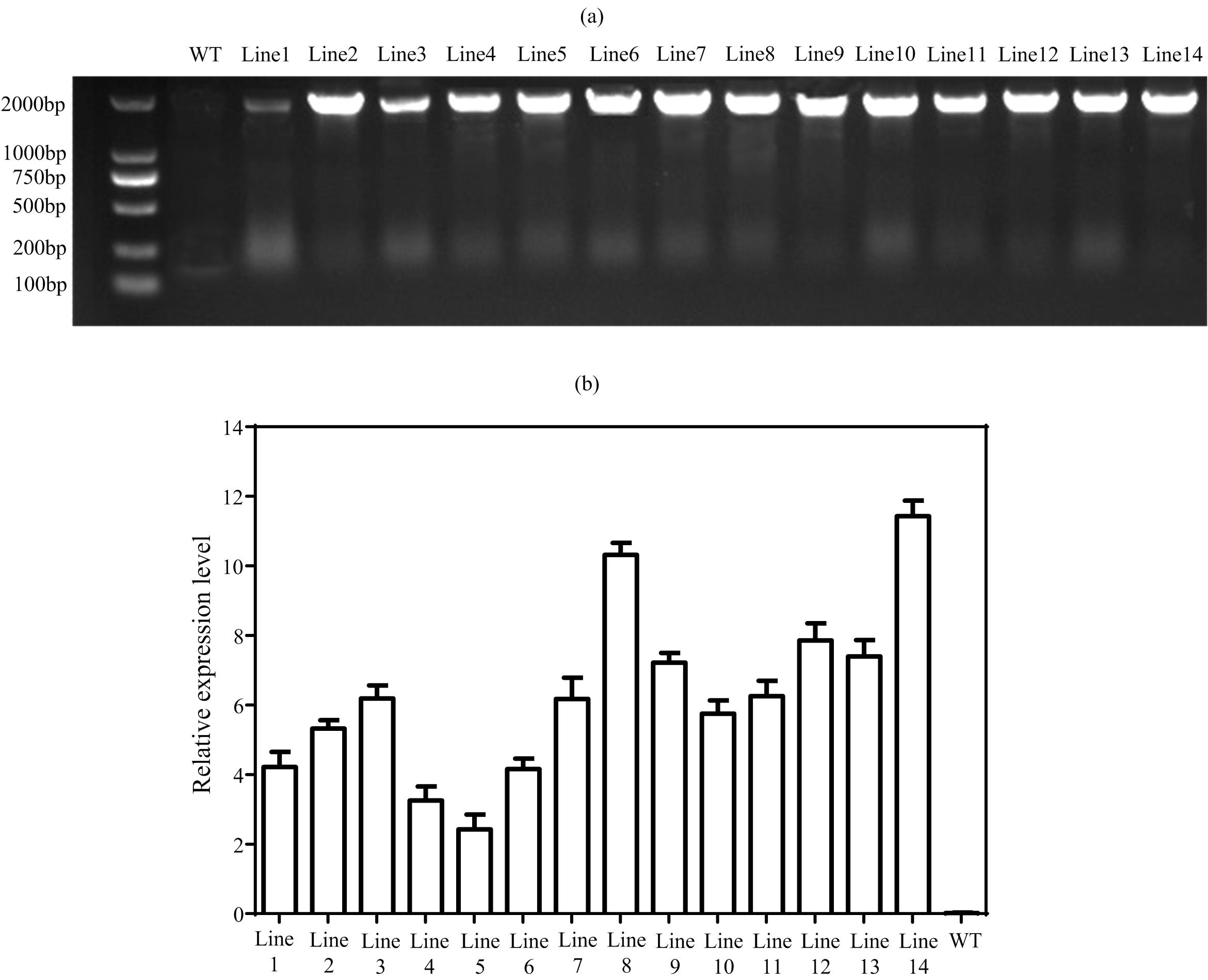

Supplement: Supplementary file 1 [file plants-10-01827-s001.zip › Figure S2.jpg]

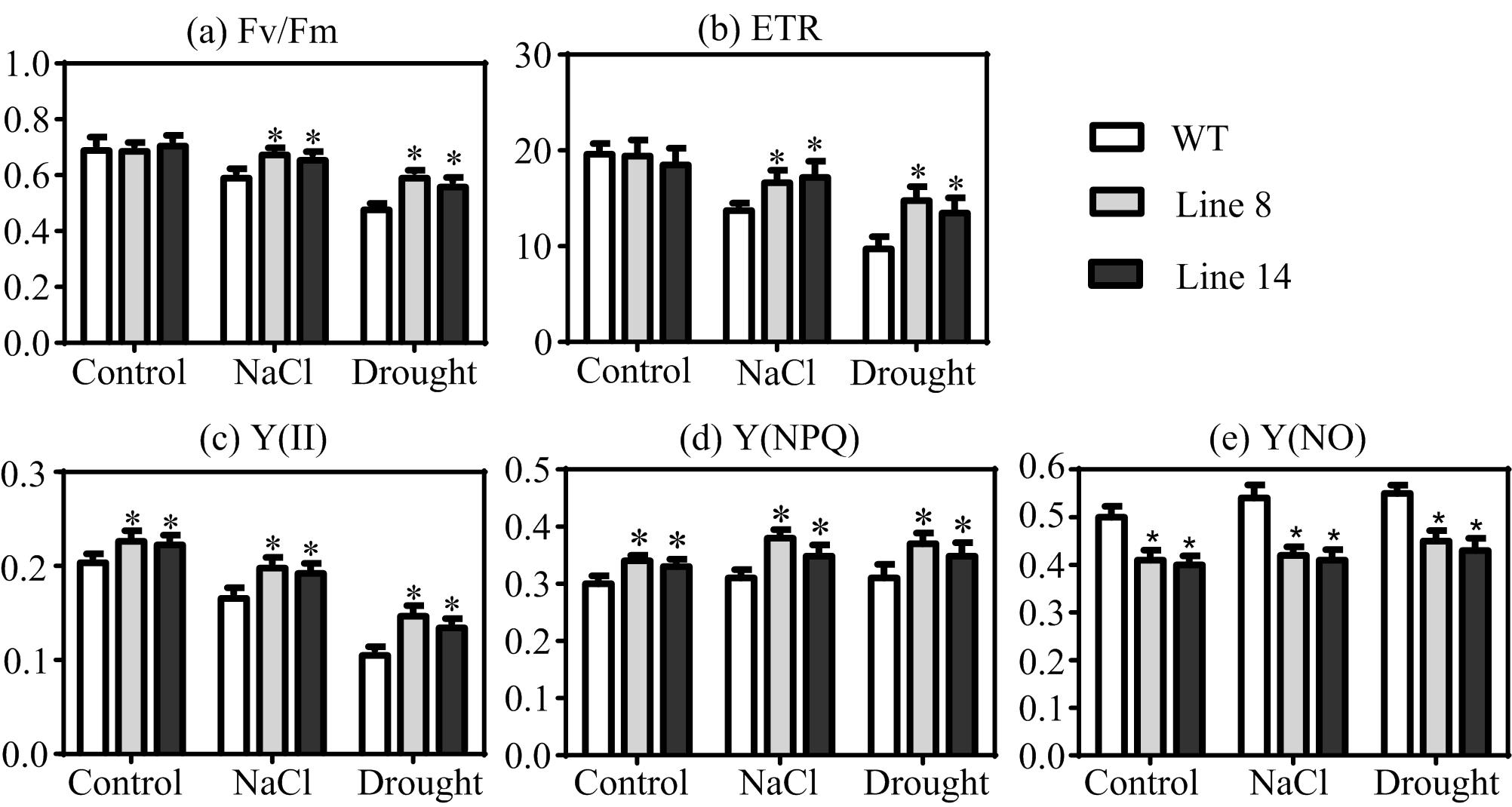

Supplement: Supplementary file 1 [file plants-10-01827-s001.zip › Figure S3.jpg]

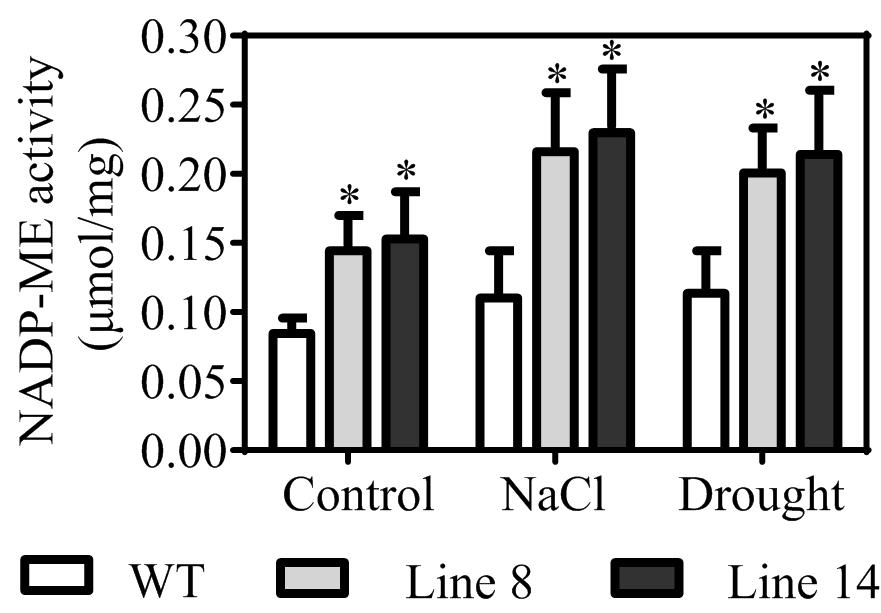

Supplement: Supplementary file 1 [file plants-10-01827-s001.zip › Figure S4.jpg]
